# Temperature and flow rate limit the optimal ex-vivo perfusion of the heart - an experimental study

**DOI:** 10.1186/s13019-020-01223-x

**Published:** 2020-07-22

**Authors:** Mohammed Quader, Juan Francisco Torrado, Martin J. Mangino, Stefano Toldo

**Affiliations:** 1 Department of Surgery, Virginia Commonwealth University, Richmond, VA USA; 2 Department of Cardiology, VCU Pauley Heart Center, Virginia Commonwealth University, Box 980281, Richmond, VA 23298 USA; 3 Department of Cardiology, Clinic Hospital, School of Medicine, Republic University, Montevideo, Uruguay

**Keywords:** Myocardial oxygen consumption, Critical coronary flow, Organ preservation, Heart transplantation, Machine perfusion system, Crystalloid solution

## Abstract

**Background:**

Ex-vivo heart perfusion can be utilized to study a variety of physiologic and molecular pathways in a controlled system outside of the body. It can also be used in clinical settings such as for organ preservation before transplantation. Myocardial oxygen consumption (MVO_2_) correlates with energy production in the myocardium and can also be used to determine the balance between the oxygen supply and demand of the perfused heart. This study sought to determine an ex-vivo perfusion rate that matches the metabolic demands of the heart according to different temperatures and solution compositions (with and without the addition of erythrocytes), a flow below which the supply of oxygen is not sufficient to maintain an aerobic state of the perfused heart (“D_CRIT_”).

**Methods:**

Under general anesthesia, rat hearts were procured and preserved by perfusing with the University of Wisconsin Belzer machine perfusion system (UW Belzer MPS) solution saturated with 100% O_2_. The key elements of this solution include supraphysiological potassium (to stop the heartbeat and reduce the cellular metabolic demand), starch, gluconate and mannitol (to maintain cell wall integrity), glucose (to sustain basal metabolism), and glutathione (to scavenge free radicals). Three groups of rat hearts (*n* = 7) were randomly allocated to be perfused at 15 °C, 22 °C or 37 °C, at a varying flow index (FI) starting from a minimum of 380 mL/min/100 g to less than 50 mL/min/100 g, decreasing by 50 mL/min/100 g at 10 min intervals while measuring the MVO_2_ at each FI. Lactate was measured from coronary sinus samples to determine the onset of tissue hypoxia/anaerobic state.

**Results:**

The D_CRIT_ at 15 °C was 99.9 ± 4.9 mL/min/100 g; however, at 22 °C and 37 °C we could not reach a D_CRIT_. The myocardial oxygen demand could not be met at 22 °C and 37 °C with the maximum FI above 380 mL/min/100 g even when erythrocytes (10% V/V) were added to the solution. At 15 °C, the production of lactate was evident only below the D_CRIT_, while at 22 °C lactate production was present at all flow indices.

**Conclusions:**

Determining the D_CRIT_ for optimal ex-vivo perfusion of the heart is necessary to ensure adequate tissue oxygenation and limit anaerobic state. Temperatures employed above 15 °C limit the efficient ex-vivo perfusion preservation of heart with the UW Belzer MPS solution.

## Background

The myocardial oxygen consumption (MVO_2_) correlates with energy utilization from the cells [1, 2]. Therefore, during a steady-state, MVO_2_ is an indirect measure of the total energy utilization of the heart [1]. In a beating heart, MVO_2_ is primarily determined by heart rate, wall tension and contractility [3]. Experimental studies have shown that MVO_2_ of the arrested heart is approximately 20% of the contracting heart. This is attributed to the basal myocardial oxygen requirements needed to maintain critical membrane functions and basal metabolism [4]. Ex-vivo organ perfusion is an ideal experimental setting that allows the study of MVO_2_ in different controlled conditions, such as a beating or resting heart and at different temperatures. Since the heart will decrease its energy utilization if perfused sub-optimally (anaerobic state), measuring the MVO_2_ in itself, may not provide with certainty whether the oxygen demand of the heart is met or not. Determining the critical flow rate (D_CRIT_) above which the O_2_ demand of the heart is met is of paramount importance to prevent the state of myocardial hypoxia and for better organ preservation.

Classic perfusion preservation is a method of preserving organs ex-vivo prior to re-implantation in a recipient [5, 6]. The goals of perfusion preservation are to preserve cellular adenosine triphosphate (ATP) stores by reducing cell metabolism such as with hypothermia, along with supplying adequate oxygen delivery and nutrients, to maintain oxidative phosphorylation [5, 6]. Temperature is one of the main parameters that determine the oxygen solubility in the perfusion solution. Oxygen solubility is higher at lower temperatures [5, 6]. In addition, since temperature regulates the cellular metabolic rate, the D_CRIT_ varies by the temperature [7]. Despite the importance of the D_CRIT_, there is little to no attention paid to the flow index (FI) to be used during organ preservation or other ex-vivo experimental perfusion conditions [8]. In an optimal ex-vivo heart preservation condition the metabolic rate is reduced and adequate oxygen is delivered to maintain an aerobic state.

The objective of this study is to define the D_CRIT_ of the rat heart perfused with University of Wisconsin Belzer machine perfusion system (UW Belzer MPS) solution at three temperatures.

## Methods

### Experimental model

This experimental study investigated the effect of different temperatures on the critical flow index (D_CRIT_) of isolated rat hearts in the experimental setting of a solution-perfused Langendorff apparatus. In addition, for those perfusing conditions that could not reach the D_CRIT_ (i.e., higher temperatures), we tested whether the addition of red blood cells may improve the oxygen delivery capacity in this setting and allow to match the oxygen demands of the myocardium.

### Heart procurement

Adult male Sprague Dawley rats were selected for these experiments. A total of 21 rats were divided into three temperature groups (15 °C, 22 °C, and 37 °C). General anesthesia was achieved by administering intramuscular Ketamine-Xylazine (100–10 mg/kg), followed by endotracheal intubation and systemic heparin (500 U), which was administered into the tail vein.

Following a bilateral thoracotomy, the inferior vena cava (IVC) was cannulated with a 21G phlebotomy catheter, secured and left in place to be used for coronary sinus sample collection during the perfusion phase. The hearts were explanted and cleaned from the extracardiac tissue, leaving the inferior vena cava and the catheter in place. Next, a cannula was placed and secured into the ascending aorta. The heart mass was determined by subtracting the weight of the of the cannulas from the weight of the cannulated heart. The average heart weight was 1.22 ± 0.05 g. To increase the oxygen carrying capacity of the solution, two additional groups of hearts were perfused (22 °C and 37 °C) with the MPS solution containing purified erythrocytes. Platelet rich plasma and erythrocytes were separated from rat blood by centrifuging heparinized blood at 400 x *g*. The erythrocyte rich phase was washed in phosphate buffered saline, centrifuged again at 1200 x *g* and the purified erythrocytes were added at a 10% V/V. The experiments were conducted under the updated guidelines of the “Guide for the care and use of laboratory animals” [9]. The study protocol was approved by the Virginia Commonwealth Institutional Animal Care and Use Committee.

### Machine perfusion system

**Fig. 1:**
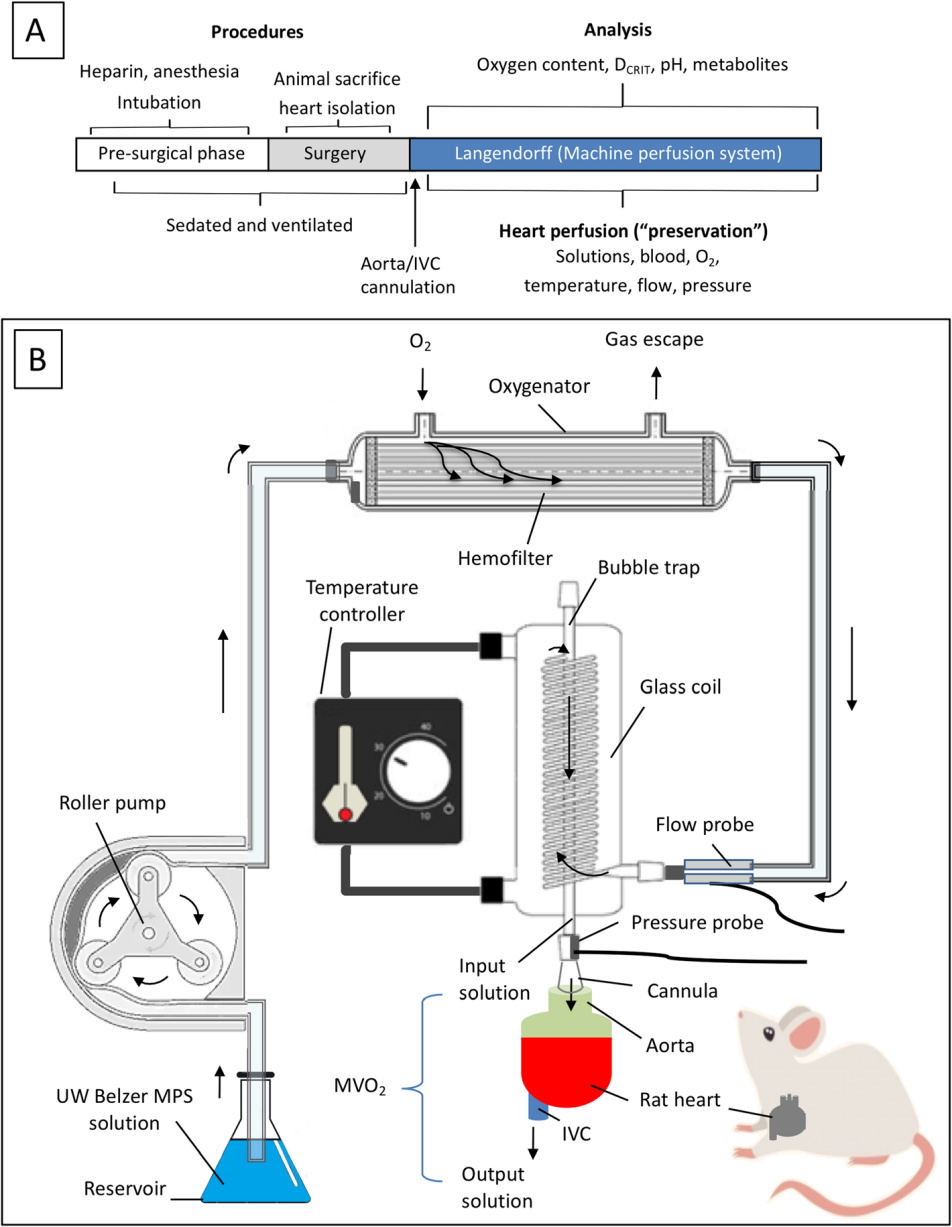
Simplified schema of the experimental design and protocol. Panel **a**. Experimental protocol employed to determine the ex-vivo critical flow index (D_CRIT_) of isolated rat hearts according to changes in the composition and temperature of the perfusate. Panel **b**. The machine perfusion apparatus is primed with oxygenated UW Belzer MPS solution at the desired temperature by using a recirculating temperature controller and glass heat exchanger coil. Hearts are attached to the apparatus, and antegrade coronary flow is initiated via the aortic cannula at a controlled flow index (FI) by using a roller pump. Oxygen transfer to the perfusate is maintained by passing the solution through a pediatric hollow fiber hemofilter with a continuous oxygen sweep delivered across the outer compartment. Flow rate, flow pressure, and temperature of perfusate are monitored continuously. Input (aortic) and output solution (inferior vena cava) samples are collected serially to assess variables of interest

The machine perfusion (MPS) system was designed to perfuse the heart at varying flow rates. The UW Belzer MPS solution (Belzer MPS®, Bridge to Life Ltd., Columbia, SC) was placed into a preservation solution reservoir and pushed into the system using a roller pump to deliver low volumes of solution (Fisher Scientific, Hampton, NH). The composition of the UW Belzer MPS solution is reported in Table 1. This is an isotonic solution that has a supraphysiological potassium concentration that maintains heart in diastolic arrest. It has been designed to reduce the cellular metabolic demand, reduce edema and maintain cell wall integrity, sustain basal metabolism and scavenge free radicals. The flow rate was measured using an in-line flow probe (AD instrument), following passage through an oxygenator (adapted from a Hemofilter D150, Medica, Monza, Italy). Placement of a cooling/heating chamber in front of the heart cannula enabled collection of sample solution with a 3-way stop-cock before entering the heart (input solution). The aortic cannula perfused the heart while the inferior vena cava cannula collected the perfusate exiting the coronary sinus (output solution). A simplified schema of the experimental protocol and MPS is represented in Fig. 1.

**Table 1:** Composition of the UW Belzer MPS solution

CONSTITUENT	CONCENTRATION
Adenine (free base)	5 (mmol/L)
Calcium Chloride (dihydrate)	0.5 (mmol/L)
Glucose	10 (mmol/L)
Glutathione (reduced)	3 (mmol/L)
HEPES (free acid)	10 (mmol/L)
Hydroxyethyl Starch	50 (g/L)
Magnesium Gluconate (anhydrous)	5 (mmol/L)
Mannitol	30 (mmol/L)
Potassium Phosphate (monobasic)	25 (mmol/L)
Ribose, D(−)	5 (mmol/L)
Sodium Gluconate	80 (mmol/L)
Sodium Hydroxide	0.7 (g/L)
Sterile Water for Injection	To 1 L final volume

The partial pressure of O_2_ in the input MPS solution (PaO_2_) was above 700 mmHg at 15 °C (mean PaO_2_ 724 ± 24), above 650 mmHg at 22 °C (mean PaO_2_ 766 ± 27), and above 450 mmHg at 37 °C (mean PaO_2_ 526 ± 55). The differences in these partial pressure values are due to the effect of temperature on oxygen solubility in a non-blood based solution. Flow values were expressed as FI as a standard measure for organ perfusion. All hearts in this study were set at an initial FI of at least 380 mL/min/100 g, equivalent to a flow rate of at least 3.8 ml/g/min. Input and output perfusate samples were collected 10 min from the beginning of the perfusion, using ice-cold 1 mL syringes. The syringes were capped with parafilm, placed on ice and rapidly analyzed (< 1 min) with the ABL-800 blood gas analyzer (Radiometer, Copenhagen, Denmark). Rigorous steps such as, pre-cooling, sealing and rapid reading, were necessary to ensure reliable readings from the samples. The perfusate flow rate was decreased by approximately 40–60 mL/min/100 g, or 0.4–0.6 ml/g/min, every 10 min after each measurement, until reaching the lowest perfusion rate allowed by the pump (30–40 mL/min/100 g, or 0.3–0.4 ml/g/min).

**Fig. 2:**
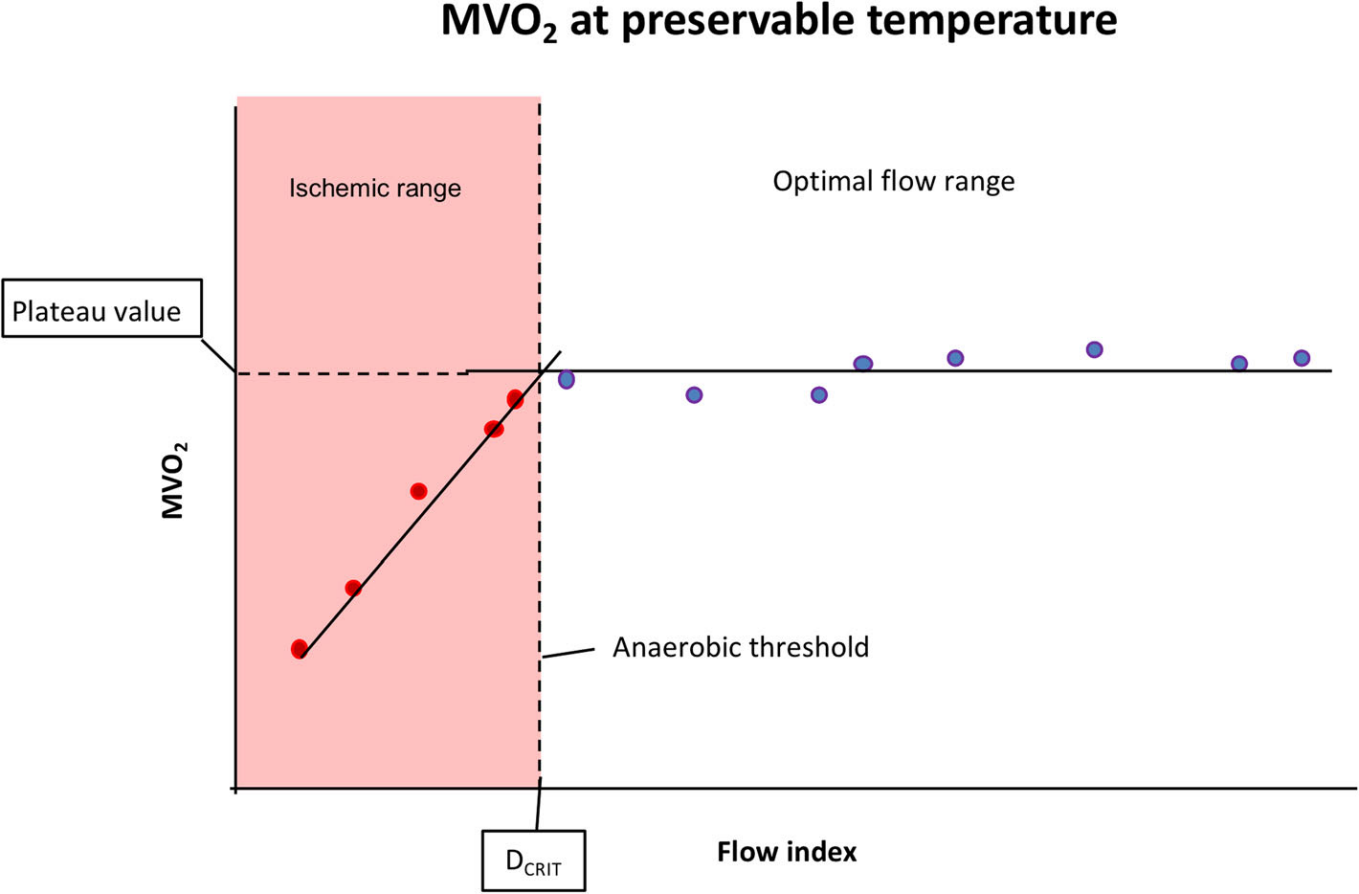
Illustration depicting the D_CRIT_ in ex-vivo heart perfusion models. The point above which oxygen extraction is flow independent is D_CRIT_. The isolated heart could not be efficiently preserved below this critical flow index since it will become hypoxic/ischemic (below “anaerobic threshold”) leading to lactic acidosis, impaired microcirculation and tissue damage. The “plateau value” (maximum MVO_2_ in relation with the temperature of the perfusate) is achieved once the critical flow value is exceeded ensuring optimal perfusion conditions

Flow rate and temperature of perfusate were monitored continuously with a Power Lab data acquisition system (AD Instruments, Denver, CO). MVO_2_ values were calculated at each FI using the Fick equation [(CaO_2_-CvO_2_) x flow / heart weight], where CaO_2_ and CvO_2_ represent the concentration of O_2_ in the input and output solution, respectively. MVO_2_ and FI values were plotted in a graph. Initiation of the anaerobic phase is represented by the corresponding FI (D_CRIT_) when MVO_2_ becomes flow limited. The exact value of the D_CRIT_ was determined from the best fit lines of the points in the plateau phase (aerobic, above the D_CRIT_) and the line derived in the flow limited part of the curve (anaerobic, below the D_CRIT_) (Fig. 2) [10]. The FI corresponding to the point when MVO_2_ drops to become flow limited represents D_CRIT_. However, when the MVO_2_ always remained flow limited (never reached a stable plateau phase) we could not determine the D_CRIT_ for the FI used.

### Lactate measurement

Lactate levels were measured in the perfusate samples using the ABL-800 blood gas analyzer at all FI. Lactate production was compared by using area under the curve for Lactate and FI for heart groups perfused at 15 °C and 22 °C.

### Statistical analysis

Values were expressed as the mean value and standard error of the mean (SEM). The sample size was not determined a priori. The statistical analysis was performed using Statistical Package for Social Sciences (SPSS version 24.0 for Mac, Chicago, IL). Differences between groups were assessed using two-tailed Student’s T test for unpaired data. Linear regression analyses were used to assess the relationship between MVO_2_ and FI. Linear equations for the lines of best fit were used to determine the D_CRIT_ if a plateau of MVO_2_ was reached. *P* < 0.05 indicates significant statistical differences.

## Results

### Effects of temperature on the FI/MVO_2_ relationship

**Fig. 3:**
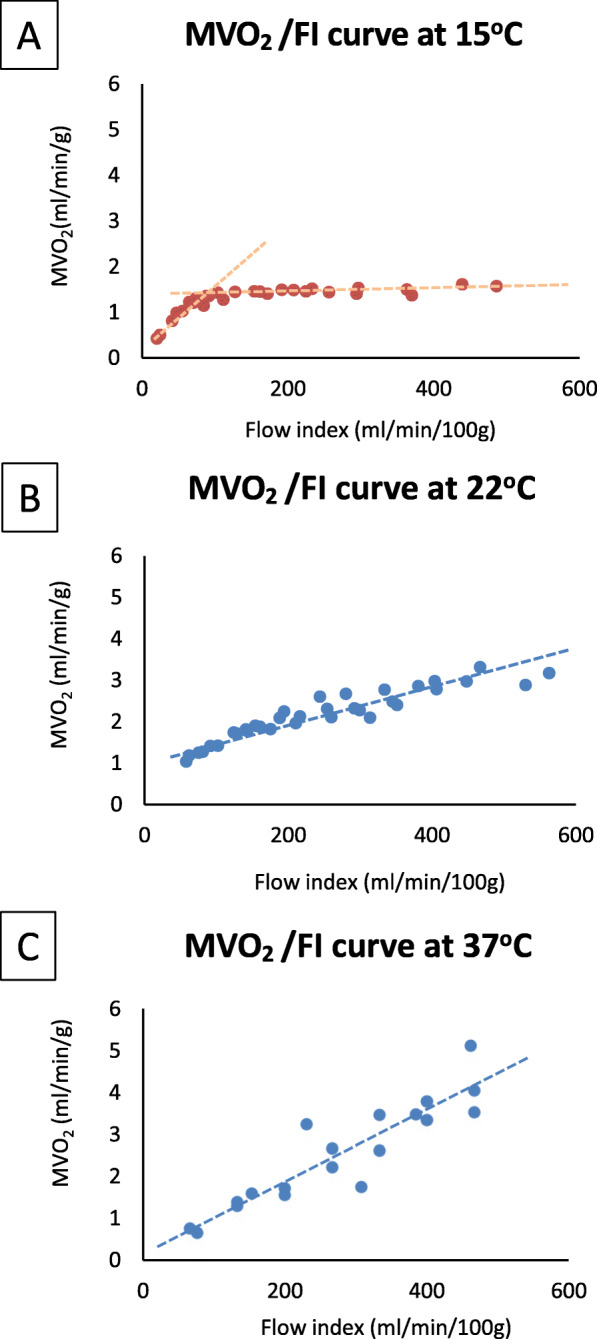
**MVO**_**2**_**/FI relationship at 15 °C, 22 °C and 37 °C.** The values of MVO_2_ and FI of different hearts perfused at 15 °C (panel **a**), at 22 °C (panel **b**) and 37 °C (panel **c**) were interpolated together. The MVO_2_ was calculated for each single heart

**Fig. 4:**
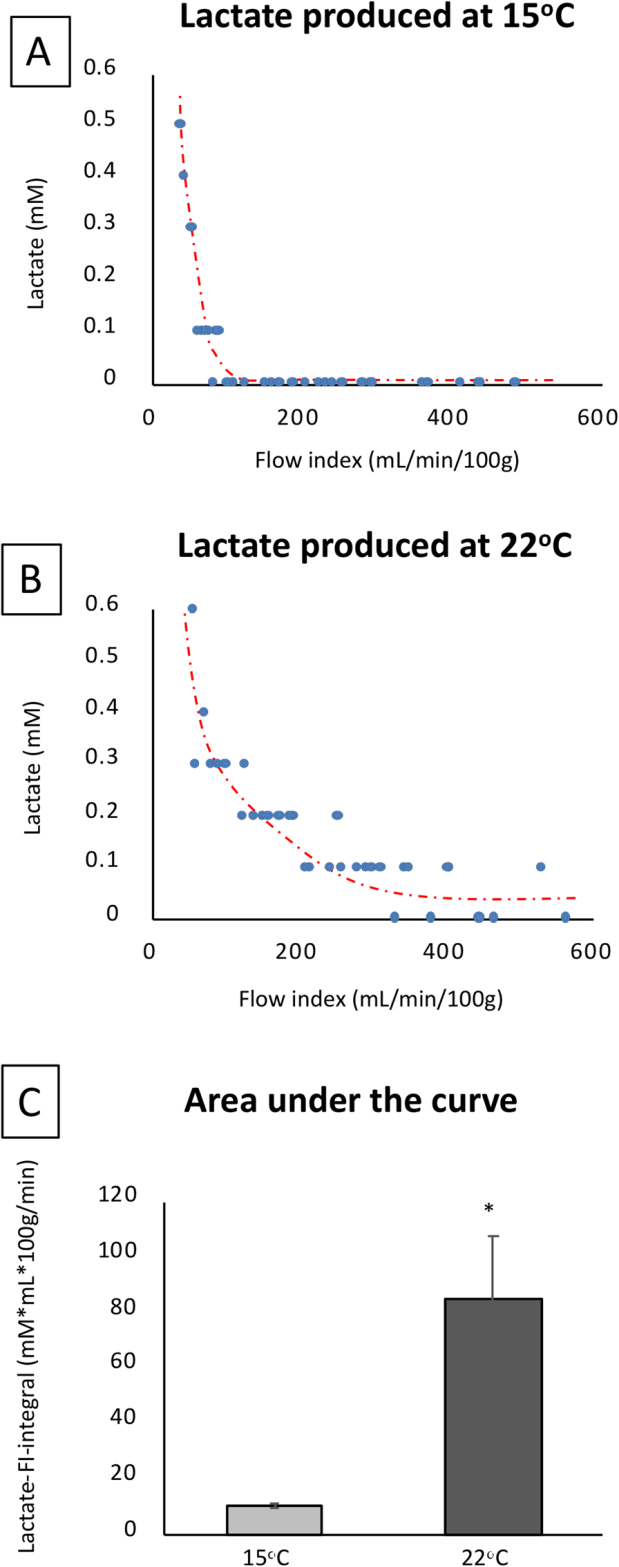
**Lactate production.** The graphs show the levels of lactate measured at each flow index analyzed during perfusion at 15 °C (panel **a**) and 22 °C (panel **b**). Panel **c** shows the value of the area under the curve (AUC) of lactate over the different range of flow indexes used and reported as mean ± SEM. *p < 0.02 for 15 °C vs 22 °C. The red dashed lines in panel **a** and panel **b** show the different trend of distribution of the plotted points in the two condition

Figure 3 shows the relationship between the FI and MVO_2_ at 15 °C, 22 °C and 37 °C using UW Belzer MPS solution. We determined that at 15 °C the D_CRIT_ is 99.9 ± 4.9 mL/min/100 g of myocardial tissue, or 1.0 ml/gram heart tissue. At this temperature, the MVO_2_ above the D_CRIT_ was 1.49 ± 0.05 mL/min/g with no further increase in MVO_2_ with higher FI. This data shows that during perfusion with UW Belzer MPS solution at 15 °C the oxygen need of the heart can be met by perfusing above the D_CRIT_. For the 22 °C and 37 °C temperatures, the relationship between MVO_2_ and FI was linear and never reached a steady-state. This data was corroborated by the analysis of the lactate levels measured in the output solution at 15 °C and 22 °C (Fig. 4). At 15 °C, there was no lactate production in the steady-state and flow-independent phase (above the D_CRIT_), while lactate levels increased consistently when the flow dropped below the D_CRIT_. On the other hand, at 22 °C there was lactate production in most of the samples at every FI analyzed. Considering these observations, lactate production in the solutions at 37 °C was predictable and not necessary to be analyzed. The difference in lactate levels between the two temperatures was significant, as shown by the analysis of the area under the curve for the lactate production at the different FI investigated (Fig. 4).

### Addition of erythrocytes to the MPS solution

**Fig. 5:**
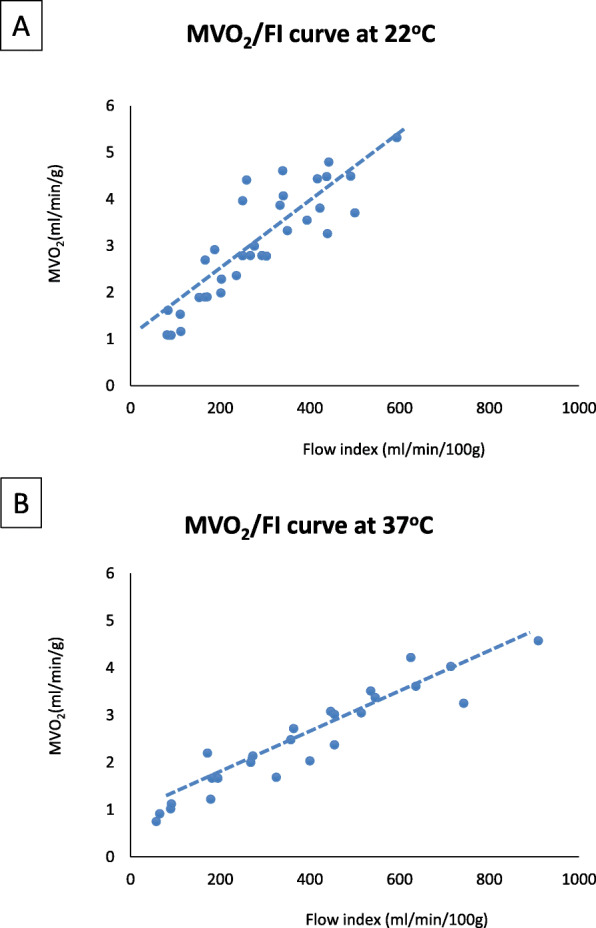
MVO_2_/FI relationship with UW Belzer MPS solution supplemented with erythrocytes at 22 °C and 37 °C. Relationship of MVO_2_ and FI of different hearts perfused with UW Belzer MPS solution supplemented with erythrocytes (10% V/V) at 22 °C (panel **a**) and 37 °C (panel **b**)

In separate groups of hearts, we added erythrocyte to the UW Belzer MPS solution at 22 °C and 37 °C, to determine whether adding an oxygen carrier could improve oxygen delivery to the heart perfused in the MPS system. Table 2 shows the input O_2_ concentration (CaO_2_) and the amount of hemoglobin measured in the perfusion solution. Despite the increase in O_2_ concentration in the input solution, adding the erythrocytes was insufficient to establish an aerobic metabolism, since the hearts did not reach a D_CRIT_ at the FI we analyzed (Fig. 5).

**Table 2:** Input O_2_ concentration (CaO_2_) of the MPS solutions with and without the addition of erythrocytes

	22 °C MPS solution	22 °C MPS solution + Erythrocytes	37 °C MPS solution	37 °C MPS solution + Erythrocytes
**CaO**_**2**_**(mL/dL)**	2.17 ± 0.07 *^,^^	3.14 ± 0.27	1.58 ± 0.16 *	2.69 ± 0.30
**Hb (g/dL)**	N/A	0.79 ± 0.20	N/A	0.73 ± 0.19

Data are expressed as mean ± SEM. *p < 0.05 for MPS solution vs MPS solution + Erythrocytes; ^*p* < 0.05 for 22 °C vs 37 °C.

Abbreviations: *MPS* machine perfusion system, *CaO*_*2*_ input O_2_ concentration, *Hb* hemoglobin, *N/A* not applicable

## Discussion

MVO_2_ is a useful parameter measured to assess energy production and utilization by the myocardial tissue [1, 2]. In this study, we have used the MVO_2_ to determine the conditions of adequate myocardial perfusion and preservation. We investigated the relationship that exist between temperature, FI and MVO_2_ in hearts perfused with UW Belzer MPS solution with the goal to determine the critical flow, or D_CRIT_, above which the myocardial oxygen demand is met. This study was performed because we felt that most of the studies performed using ex vivo perfusion are designed based on empirically determined observations that may underestimate the importance of perfusion flows, pressures or temperatures used. Such parameters may in fact limit the optimal oxygen delivery to the heart, which in turn may alter the tissue response to injury and or therapeutic treatments. Prior work has been performed to determine the D_CRIT_ in other organs [11, 12]. Those studies were performed using blood based perfusate in normothermic conditions. However, to our knowledge, ours is the first of such studies determining the Dcrit in the normal heart in different conditions.

We used a machine perfusion system model in combination with a cardioplegia (UW Belzer MPS solution) to exert control on all the parameters that may affects the oxygen use in the myocardium. An arrested heart has an 80% MVO_2_ lower compared to the contracting heart [4]. Interestingly, we found that the oxygen demand of a non-beating rat heart is met at a temperature of 15 °C using a flow above 100 mL/min/100 g of tissue, or 1.0 ml/gram, but not when perfusing the rat heart at 22 °C or higher temperatures with UW Belzer MPS solution. This result is particularly important because it shows that perfusion of the rat heart with UW Belzer MPS solution at temperatures of 22 °C and higher is flow limited and the heart produces lactate at the higher FI (i.e., perfusing conditions below anaerobic threshold). We used erythrocytes to increase the oxygen delivery to the heart and, interestingly, the MVO_2_ did not change in this condition. The inability to reach the D_CRIT_ with the addition of erythrocytes could be due to insufficient amount of erythrocytes in perfusate or due to impaired oxygen delivery at the level of the microcirculation, possibly related to hemo-rheological disturbances such impaired red blood cell deformability, increased cell aggregation and vasoconstriction, all leading to microvascular occlusion [13–17].

The use of a cardioplegia, like the UW Belzer MPS solution, eliminates differences in myocardial oxygen demand due to the cardiac contractility. The UW Belzer MPS solution is a colloid solution that contains glucose, adenine, ribose and phosphates to supplement the heart with basic elements to produce energy. This solution has been developed for kidney perfusion at 4 °C. However, the UW Belzer MPS is very similar to the UW cold storage storage solution, which has been used in human heart transplantation and in experimental studies in the preclinical setting [18–20]. Due to optimization for the machine perfusion system, the UW Belzer MPS has been the solution of choice in this study. It is noteworthy that if an arrested heart could not be kept in aerobic condition above 15 °C then it is not practical to expect a reanimated heart to remain in aerobic condition while perfused with non-blood based perfusates. A blood based ex vivo perfusion model will carry sufficient oxygen to keep even a reanimated heart in aerobic condition but it has its own limitations [21, 22]. Many a times, an additional animal needed to be sacrificed to prime the blood based ex-vivo circuit, this will add cost and a need for justification for additional animals. The blood is known to hemolyze in roller pumps leading to deposition of heme in the myocardial capillaries and limiting even distribution of perfusate. Since the blood based circuit is recycled after certain duration the metabolic substrate is depleted and a continuous addition of substrate becomes necessary [23]. In view of these challenges, the majority of small animal ex-vivo heart perfusion studies were done with non-blood based perfusates. Our observations are particularly important for perfusion preservation studies, because our results show that above 22 °C the rat heart cannot be efficiently preserved with UW Belzer MPS solution, and that for a temperature of 15 °C there is a minimum FI requirement, below which the heart becomes ischemic. Our results suggest that ex-vivo perfusion of hearts need to be carefully planned, by determining the Dcrit of the heart, that most likely varies with the species or size and metabolism of the heart tested. Conditions like temperature may pose some limitation to the ex-vivo perfusion of the heart (and virtually every other organ). Some of the implications of our study can be extended to the field of organ preservation in the setting of donation after brain death (DBD) or donation after circulatory death (DCD). Cold storage of a DBD heart is associated with a risk of preservation injury, and perfusion preservation may be superior to cold storage due to the avoidance of cold ischemia [24]. Experimental studies have shown that cold storage is detrimental for DCD hearts, and perfusion preservation can be a suitable strategy for such hearts [25, 26]. Therefore, our platform can be used to define the best preservation conditions for both DBD and DCD donors.

## Limitations

We did not investigate further the relationship between the FI and MVO_2_ at temperatures lower than 15 °C. However, we can speculate that the heart can be safely perfused meeting its oxygen demand by perfusing at a FI above the D_CRIT_ defined here. In addition, the inability to reach the critical flow index of the isolated rat hearts with the addition of erythrocytes at higher perfusing temperatures could be explained by having used insufficient hematocrit; perhaps increasing more red blood cells may have allowed us to attain D_CRIT_. Again, although blood is theoretically an ideal perfusate for hearts with natural buffers and antioxidants [21], the use of blood-based perfusates in small animal ex-vivo heart perfusion studies is challenging. In addition, blood-based perfusion solutions are prone to hemolyze, for which hemoglobin equivalents or substitutes, as well as synthetic oxygen carrier-based solutions, would be attractive. Future studies using the O_2_ carriers might allow us to achieve D_CRIT_ at higher temperatures, thus eliminating the tight temperature control needed to conduct these experiments with currently available crystalloid-based perfusate. In a prior study, we utilized a modified version of the UW Belzer MPS solution, containing polyethylene glycol 20 K (instead of starch), adenosine Na-octanoate and aminoacids, where we could measure a D_CRIT_ at 22 °C [10], but not at higher temperatures (data not shown). Our results imply that during normothermic perfusions with physiological crystalloid buffers like the Krebs-Henseleit (K-H), which allows spontaneous development of the heartbeats, the heart is perfused under hypoxic/ischemic conditions. This may explain why following perfusion with the K-H buffer, the heart function steadily declines with time [27].

## Conclusions

Determining the D_CRIT_ for optimal ex-vivo perfusion of the heart is necessary to ensure adequate tissue oxygenation and limit anaerobic state. In the investigated isolated rat heart model, the use of UW Belzer MPS solutions at 15 °C allows optimal conditions for the machine perfusion system to exceed the D_CRIT_ and limit tissue damage. Temperatures employed above 15 °C limit the efficient ex-vivo perfusion preservation of the heart, even with the addition of erythrocytes to the MPS solution.

## Data Availability

All data generated or analyzed during this study are included in this published article.

## Abbreviations

CaO_2_: Input O_2_ concentration
D_CRIT_: Critical flow index
FI: Flow index
Hb: Hemoglobin
IVC: Inferior vena cava
K-H: Krebs-Henseleit
MVO_2_: Myocardial oxygen consumption
SEM: Standard error of the mean
UW Belzer MPS: University of Wisconsin Belzer machine perfusion system
